# Moroccan *Leishmania infantum*: genetic diversity and population structure as revealed by multi-locus microsatellite typing

**Published:** 2011-01-10

**Authors:** Salsabil Hamdi, Ahmad Amro, Gabriele Schönian, Meryem Lemrani

**Affiliations:** 1 Département de Recherche, Institut Pasteur du Maroc, Morocco; 2 Faculty of Pharmacy, Al-Quds University, Jerusalem, Palestine; 3 Charité Universitätsmedizin. Institut für Mikrobiologie und Hygiene, Berlin, Germany

## 

*Leishmania infantum* causes visceral and cutaneous leishmaniasis in northern Morocco. It predominantly affects children under 5 years with an incidence of 100 cases per year. Genetic variability and population structure has been investigated for 55 strains isolated from infected dogs and humans in Morocco. A multilocus microsatellite typing (MLMT) approach was used in which a MLM type based on size variation in 14 independent microsatellite markers was compiled for each strain. MLMT profiles of 10 Tunisian, 10 Algerian and 21 European strains which belonged to zymodeme MON-1and non-MON1 according to multilocus enzyme electrophoresis (MLEE) were included for comparison. A Bayesian model-based approach and phylogenetic analysis inferred two *L. infantum* sub-populations; Sub-population A consists of 25 Moroccan strains grouped with all European strains of MON-1 type; and sub-population B consists of 25 Moroccan strains grouped with the Tunisian and Algerian MON-1 strains. Theses sub-populations were significantly different from each other and from the Tunisian, Algerian and European non MON 1 strains which constructed one separate population. The presence of these two sub-populations co-existing in Moroccan endemics shed the light on the possible scenarios of multiple introduction of *L. infantum* from/to Morocco; (1) Introduction from/to the neighboring North African countries, (2) Introduction from/to the Europe. These scenarios are supported by the presence of sup-population B and sub-population A respectively. Gene flow was noticed between sup-populations A and B. Five strains showed mixed A/B genotypes indicating possible recombination between the two populations. MLMT has proven to be a powerful tool for eco-epidemiological and population genetic investigations in *Leishmania*.

